# Continuous Theta Burst Transcranial Magnetic Stimulation of the Right Dorsolateral Prefrontal Cortex Impairs Inhibitory Control and Increases Alcohol Consumption

**DOI:** 10.3758/s13415-018-0631-3

**Published:** 2018-08-21

**Authors:** Adam McNeill, Rebecca L. Monk, Adam W. Qureshi, Stergios Makris, Derek Heim

**Affiliations:** Department of Psychology, Edge Hill University, St Helens Road, Ormskirk, L39 4QP UK

**Keywords:** Inhibitory control, Disinhibition, TMS, Alcohol, Binge drinking

## Abstract

Previous research indicates that alcohol intoxication impairs inhibitory control and that the right dorsolateral prefrontal cortex (rDLPFC) is a functional brain region important for exercising control over thoughts and behaviour. At the same time, the extent to which changes in inhibitory control following initial intoxication mediate subsequent drinking behaviours has not been elucidated fully. Ascertaining the extent to which inhibitory control impairments drive alcohol consumption, we applied continuous theta burst transcranial magnetic stimulation (rDLPFC cTBS vs. control) to isolate how inhibitory control impairments (measured using the Stop-Signal task) shape *ad libitum* alcohol consumption in a pseudo taste test. Twenty participants (13 males) took part in a within-participants design; their age ranged between 18 and 27 years (*M* = 20.95, *SD* = 2.74). Results indicate that following rDLPFC cTBS participants’ inhibitory control was impaired, and ad libitum consumption increased. The relationship between stimulation and consumption did not appear to be mediated by inhibitory control in the present study. Overall, findings suggest that applying TMS to the rDLPFC may inhibit neural activity and increase alcohol consumption. Future research with greater power is recommended to determine the extent to which inhibitory control is the primary mechanism by which the rDLPFC exerts influence over alcohol consumption, and the degree to which other cognitive processes may play a role.

## Introduction

When talking about alcohol-related behaviours, “just going for one!” is a commonly expressed sentiment that all too frequently appears to precede heavy (albeit unplanned) alcohol consumption. According to such anecdotal wisdom, the consumption of alcohol may lessen self-control and undermine good intentions of engaging in restrained drinking. The (in)ability to control or suppress preponent responses, known as inhibitory control (de Wit & Richards, 2004; De Wit, 2009; Olmstead, 2006), is increasingly being recognized in the literature as both a determinant and a consequence of alcohol consumption (De Wit 2009), as well as being implicated in other behaviours that require exerting a degree of self-control (Houben, Nederkoorn, & Jansen, 2014; Lane, Cherek, Pietras, & Tcheremissine, 2004). Inhibitory control impairments have been documented in samples of alcohol-dependent individuals (Goudriaan, Oosterlaan, De Beurs, & Van Den Brink, 2006; Lawrence, Luty, Bogdan, Sahakian, & Clark, 2009), and longitudinal studies suggest that inhibitory control predicts both future alcohol consumption and alcohol-related problems (Nigg et al., 2006). Concurrently, lower levels of inhibitory control appear to be associated with heavy, hazardous and problematic drinking in nondependent samples (Christiansen, Cole, Goudie, & Field, 2012; Murphy & Garavan, 2011; Nederkoorn, Baltus, Guerrieri, & Wiers, 2009). As such, current evidence converges to implicate inhibitory control in the regulation of alcohol consumption.

Alcohol preloads (acute intoxication) have been found to result in subsequent increases in consumption (Rose & Grunsell, 2008) and to be associated with transient impairments in inhibitory control (Caswell, Morgan, & Duka, 2013; Fillmore & Rush, 2001; Rose & Duka, 2006; Weafer & Fillmore, 2012). Fluctuations in inhibitory control have been proposed to mediate the relationship between the alcohol preload and subsequent alcohol consumption (Field, Wiers, Christiansen, Fillmore, & Verster, 2010; Jones, Christiansen, Nederkoorn, Houben, & Field, 2013). Empirical evidence of the extent to which inhibitory control mediates the association between initial intoxication and continued alcohol consumption, however, is mixed. Some studies find that impairments in inhibitory control correlate with subsequent consumption (Weafer & Fillmore, 2008), while others investigating this directly find no mediation (Christiansen, Rose, Cole, & Field, 2013). A particular research challenge is to unpack the reasons why initial alcohol consumption may inadvertently lead to continued drinking (e.g., via impulsivity or craving; Rose & Grunsell 2008). Existing paradigms frequently administer alcohol to induce impaired inhibitory control and to examine how this impacts control over subsequent alcohol consumption. However, acute alcohol intoxication is also associated with a range of changes to other cognitive and psychological processes (e.g., attentional bias and motivations to drink; Fadardi & Cox (2008)), and it has therefore been difficult to disentangle the extent to which inhibitory control is implicated in the maintenance of alcohol consumption. Also in view of wide-reaching costs associated with excessive alcohol consumption (World Health Organization, 2014), more research is therefore required to examine this relationship, and to ascertain underlying neuropharmacological processes (Volkow, Koob, & McLellan, 2016).

Anticipation of reward has been associated with heightened activations in the dorsolateral prefrontal cortex (DLPFC), the medial orbital frontal cortex and activation in the ventral striatum (VS) in individuals with substance use disorders (Luijten, Schellekens, Kuehn, Machielse, & Sescousse, 2017 for a recent review). In response to alcohol consumption, *f*MRI studies point to acute decreases in the activation of neural regions associated with inhibitory control, including the DLPFC (Bjork & Gilman, 2014). Meanwhile, Positron Emission Tomography (PET) research on healthy participants suggests that moderate doses of alcohol are associated with reductions in overall brain metabolism, although metabolic increases are observed in mesolimbic regions involved in the incentive-motivational system, including the VS and nucleus accumbens (NAc) (Volkow et al., 2008). Thus, by examining the acute responses of the brain to alcohol, researchers have begun to illuminate the effects and drivers of alcohol intoxication, behavior, and cognition (Bjork & Gilman 2014; Volkow et al., 2008). However, methods, such as *f*MRI and PET, do not allow us to investigate how alcohol-related neurological changes directly influence cognitive processes and how these may, in turn, drive fluctuations in alcohol consumption.

Addressing this by enabling researchers to assess the causal links between specific regions and their functions, Transcranial Magnetic Stimulation (TMS) is a useful means of impeding particular brain areas. Existing research implicates regions of the prefrontal cortex, including the rDLPFC, in inhibitory control processes, and a recent review documents that active TMS stimulation (compared with control) to prefrontal regions is an effective means of impairing inhibitory control (Lowe, Manocchio, Safati & Hall, 2018). While this evidence implicates rDLPFC in the inhibitory control processing, the extent to which this impacts alcohol consumption has yet to be elucidated fully.

The present study used TMS to impede rDLPFC functioning to ascertain the extent to which inhibition impairments contribute to alcohol consumption. Specifically, in view of the preponderance of research impairing inhibitory control by acute administration alcohol (Caswell et al., 2013; Fillmore & Rush 2001; Rose & Duka 2006; Weafer & Fillmore 2012), we used TMS to assess directly the relationship between impaired inhibitory control and alcohol consumption, independent from the wider pharmacological effects of alcohol. A within-participant design was utilized to the test the hypothesis that TMS-induced impaired inhibitory control would result in increased alcohol consumption *ad libitum* compared with control stimulation and that impaired inhibitory control would mediate this relationship.

## Method

### Participants

Twenty participants (13 males, age between 18 and 27 years**,***M* = 20.95, *SD* = 2.74) were recruited in response to online advertisements which sought to recruit fluent English speakers aged between 18 and 49 years who regularly use alcohol and exceed recommended weekly drinking guidelines (14 units). Due to the risks associated with TMS, participants also were required to complete a medical screening form. Participants whose medical history indicated any neurological risk factors, syncopy, drugs active in the central nervous system (e.g., antipsychotics, antidepressants, or recreational stimulants), and poor levels of sleep were excluded from the study (Rossi, Hallett, Rossini, & Pascual-Leone, 2009; Wassermann, 1998). It is worth noting that the risks associated with cTBS are minimal, with only one known case of seizure as of Rossi et al. (2009). Participants who had sought help concerning their drinking or had a history of alcohol dependency also were excluded. As reimbursement for their time, participants were either awarded course credit or £12. The study received ethical review and clearance from the University’s Department of Psychology Research Ethics Committee.

### Design

A counterbalanced, within-participants design was implemented. The independent variable of TMS stimulation consisted of two levels: cTBS TMS stimulation to the rDLPFC, and control stimulation consisting of cTBS at the same intensity to the Vertex. Measures of inhibitory control and subsequent drinking were taken. Approximately 6 minutes passed between cTBS and the subsequent drinking task. This is the approximate time to complete the inhibitory control task.

### Materials

#### Questionnaires

##### *Time Line Follow Back* (TLFB: Sobell & Sobell, 1990)

Participants are required to retrospectively report their daily alcohol consumption (in units) for the previous 14 days.

##### *Alcohol Use Disorder Identification Test* (AUDIT: Saunders, Aasland, Babor, de la Fuente & Grant, 1993)

The AUDIT is a 10-item questionnaire concerning levels of alcohol consumption and its consequences. Scores range from 0-40, with scores ≥8 representative of alcohol consumption of a hazardous level.

##### *Barrett Impulsivity Scale* (BIS-11: Patton, Stanford, & Barratt, 1995)

The BIS is a multidimensional scale, consisting of three subscales; attentional, motor and nonplanning impulsiveness. BIS-11 includes 30 fixed response items (e.g., I plan tasks carefully), which are assessed on a 4-point scale (rarely/never – almost always/always). Higher scores are indicative of increased impulsivity.

#### Behavioural tasks

##### *Stop-signal task* (SST: Verbruggen, Logan, & Stevens, 2008)

The Stop Signal task consists of two concurrent tasks: a go task (75% of trials), which is a choice reaction task where participants categorise arrows on the screen based on their orientation (left or right), and a stop task (25% of trials) where an auditory tone (the stop signal) indicates that participants should inhibit their response to the go signal. Participants are required to respond as quickly and accurately as possible to the stimuli with a predetermined corresponding key. Upon hearing the auditory tone (the stop signal) participants are required to inhibit their response. After 2,000 ms, the trial will time out.

On the stop trials, tones are delivered at fixed delays (known as Stop-signal delays or SSD) of between 50 ms and 500 ms following the presentation of the go stimulus. The stop signal task uses these SSDs dynamically, based on participant performance. The *one-up one-down tracking procedure* (Logan, Schachar, & Tannock, 1997) was implemented, which adjusts the SSDs after each trial. After successful inhibition trials, the SSD increases by 50 ms, handicapping the stop signal process on the next stop signal trial. Unsuccessful inhibition trials result in the SSD decreasing by 50 ms. In accordance with the “horse race” model, the degree of difficulty in inhibiting responding increases as the delay between the go stimulus and the stop signal increases (Logan, Cowan, & Davis, 1984). Providing an outcome variable of stop-signal reaction time (SSRT). The SST was delivered using Millisecond Inquisit Lab version 4. Participants received 3 experimental blocks of 64 trials, allowing for a short break between each block, taking approximately 6 minutes to complete.

### Theta Burst stimulation procedure

Continuous theta burst stimulation (cTBS) was performed using a 70-mm figure-of-eight stimulation coil (Magstim D70^2^ Coil), connected to a Magstim SuperRapid 2 Stimulator (The Magstim Company, Carmarthenshire, Wales). This produces a magnetic field of up to 0.8 T at the coil surface. To appropriately select the TMS stimulation intensity for each participant, the resting motor threshold (rMT) for the first dorsal interosseous muscle (FDI) of the participant’s dominant hand was visually determined (Pridmore, Fernandes, Nahas, Liberatos, & George, 1998). The coil was positioned over the left or right motor cortex (for right or left-hand dominance respectively) in correspondence with the optimal scalp position (OSP). It was detected by moving the intersection of the coil in 1-cm steps around the motor hand area of the left motor cortex, while delivering TMS pulses at constant intensity. The rMT was defined as the lowest stimulus intensity able to evoke a visible finger twitch on at least five of ten trials.

cTBS was delivered over the rDLPFC. The vertex was chosen as a control site to account for non-specific effects of TMS. The approximate locations of the stimulating areas were identified on each participant's scalp by means of the 10-20 EEG System Positioning. In keeping with past research, for rDLPFC stimulation, the coil was positioned on the F4 location. Three-pulse bursts at 50 Hz repeated every 200 ms for 40s were delivered at 80% of the subject’s resting MT (equivalent to “continuous theta burst stimulation” cTBS), resulting in 600 pulses in total (Huang, Edwards, Rounis, Bhatia, & Rothwell, 2005). The coil was positioned tangentially to the scalp, at 90° from the midsagittal line, to modulate contralateral M1 excitability and interfere with cognitive functions. The coil was held by hand throughout stimulation and the exact coil position was marked by ink to ensure an accurate and consistent positioning of the coil throughout the experiment. The inhibitory effect of cTBS with this protocol lasts up to 30 minutes (Cho et al., 2010; Huang et al., 2005).

### *Ad libitum* alcohol consumption

*Ad libitum* alcohol consumption was measured by means of the Bogus Taste test. Participants were presented with three different beers (330 ml each) and asked to rate them on several dimensions of taste (e.g., bitterness and sweetness). They were informed that they could consume as much or little as they liked to complete the task successfully. *Ad libitum* consumption is measured by subtracting the remaining volume from the initial volume.

### Procedure

Participants who expressed an interest in participation were first required to complete a medical screening questionnaire to ensure they could undergo TMS, additionally affording them the opportunity to ask the researcher questions. Upon entering the laboratory, participants were required to provide informed consent and supply a Breath Alcohol Concentration (BrAC) of 0.0 mg (Lion Alcolmeter 400, Lion Laboratories, Vale of Glamorgan, United Kingdom). During the first session, participants completed a battery of questionnaires, including demographic information, the TLFB, AUDIT, and BIS-11. Participants completed the SST prior to TMS stimulation in the first session to provide a baseline measure of SSRT. The within-participant order of conditions was counterbalanced. Participants either received cTBS or control stimulation in the first session and in the second session, which took place at least 1 week later, participants completed the opposite TMS condition. In both cases, participants completed the SST post stimulation, followed immediately by the bogus taste task.

### Data Reduction and Statistical Analysis

Before calculating SSRT, trails where the reaction times were less than 100 ms and greater than 2,000 ms, and those greater than three standard deviations above the participants mean were removed. SSRT was then calculated by extracting the percentage errors (failure to inhibit response on stop trials) at each of the SSDs (50-500 ms, at 50-ms intervals), then calculating an SSRT value for each SSDs based on the reaction time (RT) distribution. Overall SSRT score was calculated by averaging the SSRT values for each of the SSDs. Impaired response inhibition is demonstrated through longer SSRT values; SSRT represents an estimate of the time required to stop initiated Go response (Band, van der Molen, & Logan, 2003). Repeated measures ANOVAs were used to analyze differences between baseline and conditions for both SSRT and GoRT and for *ad libitum* consumption following rDLPFC and control cTBS. Within-participants mediation analysis to assess the relationship between impairments in inhibitory control and *ad libitum* consumption was implements as per Montoya and Hayes (2017), using the MEMORE macro for SPSS developed by the same authors.

## Results

With regard sample characteristics, participants age and alcohol involvement descriptive statistics are comparable with previous studies investigating the effects of acute alcohol on inhibitory control (Christiansen et al., 2013; Rose & Grunsell, 2008) (Table 1). Table 1 also contains descriptive statistics for the TMS protocol, including the output required to stimulate the motor cortex (rMT) and the cTBS TMS intensity output.

**Table 1:** Descriptive statistics of participant characteristics

	*M*	*SD*
Age	20.95	2.74
TLFB (UK units)	39.60	35.83
AUDIT	11.75	4.40
BIS Total	64.20	10.83
Attentional BIS	16.70	4.23
Motor BIS	24.75	4.64
Nonplanning BIS	23.50	4.92
rMT (%)	65.90	11.07
cTBS intensity (%)	52.80	8.79

TLFB = Timeline follow back; 14-day alcohol consumption in UK units. AUDIT = Alcohol Use Disorder Identification Test, scores >8 indicative of hazardous drinking. BIS = Barratt Impulsivity Scale. Attentional, motor and non-planning BIS are subscales of BIS. RMT = resting motor threshold. cTBS = continuous Theta Burst Stimulation

**Fig. 1:**
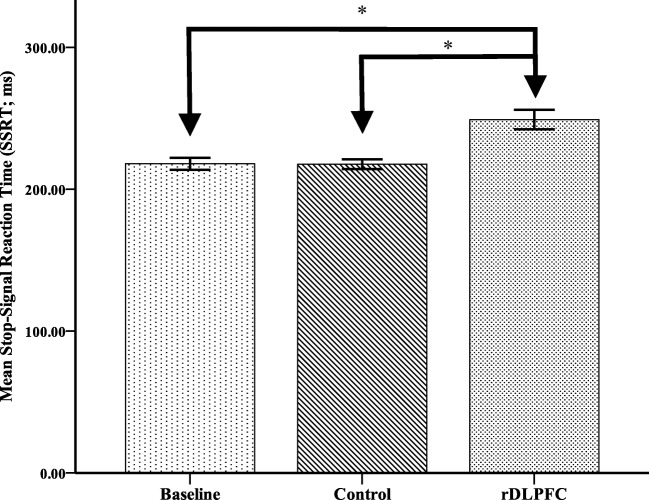
Mean stop signal reaction times (SSRT) in milliseconds and standard error bars for baseline, and following continuous theta burst transcranial magnetic stimulation to the rDLFPC and control. * *p* < .001

**Fig. 2:**
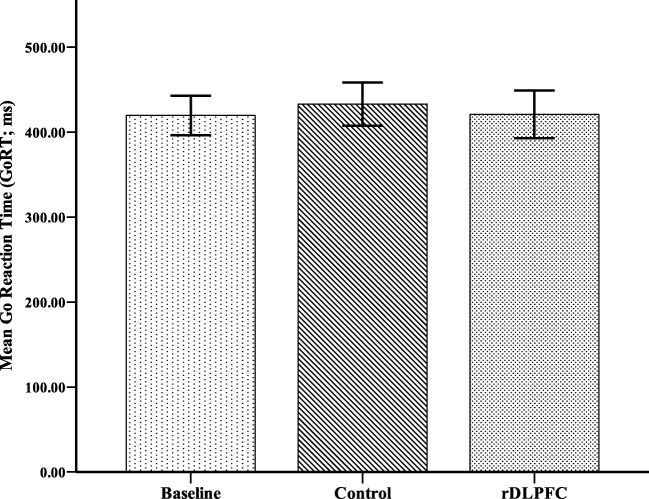
Mean go reaction times (GoRT) in milliseconds and standard error bars for baseline, and following continuous theta burst transcranial magnetic stimulation to the rDLFPC and control. * *p* < .001

A repeated-measures Analysis of Variance (ANOVA) was conducted to investigate the effects of stimulation on inhibitory control as measured by stop-signal reaction time (SSRT). A main effect of stimulation was found (*F*(2, 36) = 16.70, *p* < .001, η$\frac{2}{p}$ = 0.47). Planned comparisons revealed that while there was a significant increase in SSRT found postactive stimulation (*M* = 249.97, *SD* = 31.40; *F*(1, 18) = 18.58, *p* < 0.001, η$\frac{2}{p}$ = 0.51), there was no significant difference between baseline SSRT (*M* = 217.83, *SD* = 19.41) and postcontrol stimulation (*M* = 217.64, SD = 15.48; *F*(1, 18) = 0.003, *p* = .96, η$\frac{2}{p}$ = 0.00). This suggests the active TBS to the rDLPFC resulted in significant impairments to inhibitory control (Figure 1). A further repeated-measures ANOVA was undertaken to assess if stimulation resulted in changes in go reaction times (RT), revealing no significant differences (*F*(2, 34) = 0.41, *p* = .67, η$\frac{2}{p}$ = 0.02; Figure 2).

**Fig. 3:**
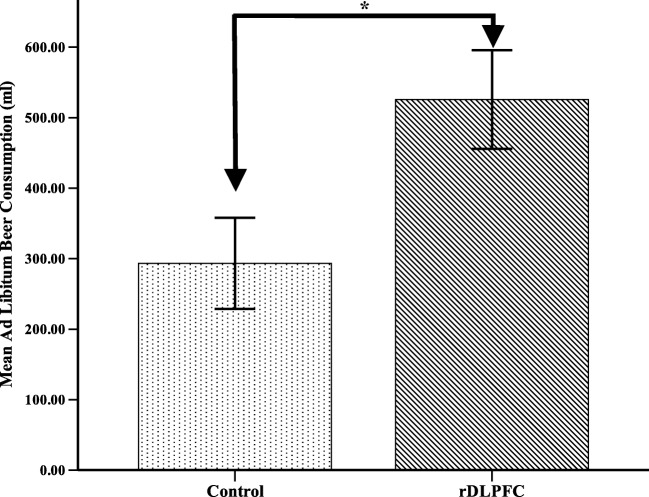
Mean *ad libitum* beer consumption (ml) and standard error bars following continuous theta burst transcranial magnetic stimulation to the rDLFPC and control. * *p* < .001

A final repeated-measures ANOVA was used to determine whether there was an effect of cTBS stimulation on *ad libitum* alcohol consumption. Results showed that participants consumed significantly more beer following active stimulation (*M* = 525.70, *SD* = 313.29) compared with postcontrol stimulation (*M* = 293.40, *SD* = 289.56; *F*(1, 19) = 19.22, *p* < 0.001, η$\frac{2}{p}$ = 0.50; Figure 3).

**Fig. 4:**
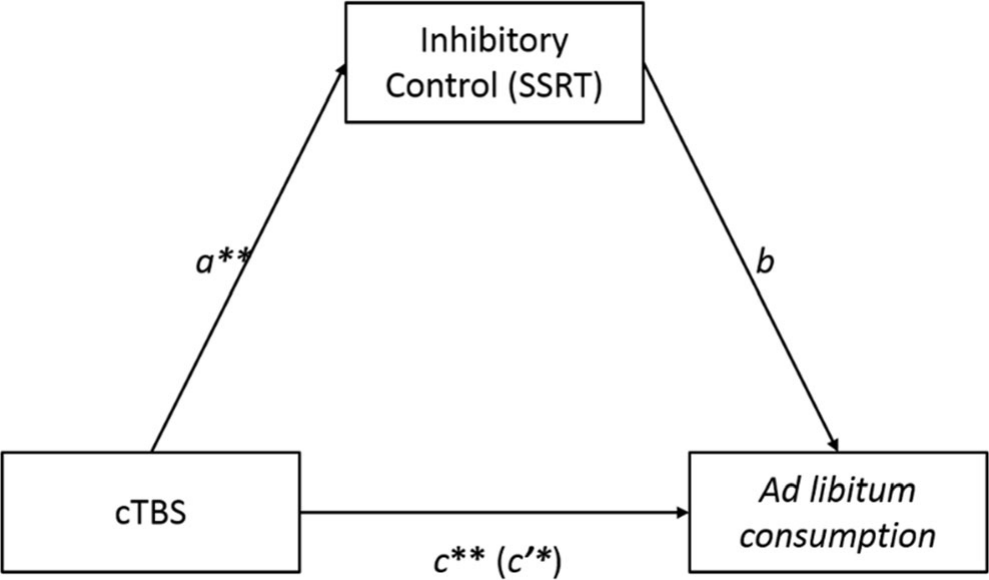
Path-analytic mediation model assessing whether impairments in inhibitory control mediate the relationship between continuous theta burst transcranial magnetic stimulation (cTBS) and *ad libitum* consumption. Significant pathways are denoted by * *p* < .01 ** *p* < .001

A within-participants mediation analysis was undertaken using the MEMORE macro for IBM SPSS (Montoya & Hayes, 2017) to test whether impairments in inhibitory control mediate changes in *ad libitum* alcohol consumption (Figure 4). Overall, the analysis showed no significant mediated pathway. The analysis revealed a significant direct effect (*c*) of cTBS on *ad libitum* beer consumption (*c*_*1*_ = 232.30, *t*(19) = 4.38, *p* < 0.001, 95% CI [121.38, 343.22]). A significant pathway *a* was also found (*a*_1_ = −31.43, *t*(19) = −4.38, *p* < 0.001, 95% CI [−46.45, −16.42]), confirming the effect of stimulation on inhibitory control. However, the *b* pathway was insignificant (*b*_1_ = 2.08, *t*(19) = 0.95, *p* = 0.36, 95% CI [−2.57, 6.75]). Furthermore, a significant indirect pathway (*c’*) was found (*c’* = 297.96, *t*(19) = 3.38, *p* < 0.01, 95% CI [112.13, 483.79]). However, because the *b* pathway in the current model was insignificant, the indication of the current findings is that impairments to inhibitory control do not mediate subsequent *ad libitum* consumption. Post-hoc Monte Carlo Simulation power analysis, running 1,000 simulations, revealed that to achieve a power of 0.80 an *N* of 200 is required.

## Discussion

Using TMS to impede the functioning of the prefrontal cortex, the current study tested the hypothesis that inhibitory control impairments mediate the relationship between cTBS to the rDLPFC and alcohol consumption. Results indicate that active (relative to control) stimulation impaired inhibitory control and increased alcohol consumption. This suggests that the rDLPFC is important in the regulation and maintenance of alcohol consumption. However, contrary to previous suggestions (Field et al., 2010; Jones et al., 2013), the current study did not yield support for the notion that impairments in inhibitory control mediate the relationship between initial and continued alcohol consumption. Our findings therefore indicate that while the rDLPFC appears to be implicated in the maintenance of alcohol consumption and impaired inhibitory control, other executive functions and psychological processes may also play a role in elevated alcohol consumption following initial intoxication.

A strength of the current study was that we were able to instigate behavioral change in terms of actual alcohol consumption by transiently impairing the rDLPFC using TMS. Previous research investigating the extent to which alcohol undermines people’s ability to exert control over behaviors has tended to rely on administering alcohol to individuals as the means of impeding behavioral control (Caswell et al., 2013; Fillmore & Rush 2001; Rose & Duka 2006; Weafer and Fillmore 2012). This work has been important in documenting the effects of acute intoxication on attentional bias (Weafer & Fillmore, 2013), executive functioning (Christiansen et al., 2013) and risk-taking (Lane et al., 2004). However, in view of findings indicating that acute alcohol exposure impacts wider executive and psychological functions (Field et al., 2010), to date it has been difficult to disentangle the relative contribution of inhibitory control to the continuation of alcohol consumption following initial intoxication. By using TMS to isolate inhibitory control impairments at the neurological level from pharmacological effects of alcohol, our study implicates temporally induced changes to the rDLPFC and inhibitory control in heightened alcohol consumption.

Our findings suggest that there was an association between stimulation of rDLPFC and impaired control and alcohol consumption, respectively. This provides support for research implicating the DLPFC in alcohol consumption (Volkow et al., 2008) as well as appetitive behaviours (Jansen et al., 2013; Lowe, Vincent, & Hall, 2017) more generally. Using, PET, Volkow et al. (2008), for example, found reduced activity in prefrontal regions following alcohol consumption. Our findings add to this body of work by causally implicating activity in prefrontal regions with alcohol consumption behaviours. In conjunction with previous work, our findings suggest that applying TMS to the rDLPFC may inhibit neural activity and increase alcohol consumption. In light of research suggesting that left prefrontal regions are also associated with impairments in inhibitory control (Lowe et al., 2018) and appetitive craving (Lowe, Hall & Staines, 2014), future research also should examine the role of the lDPFC in alcohol consumption.

The current study found no direct effect of inhibitory control on alcohol consumption, and findings indicate that the association between cTBS of the rDLPFC and consumption did not appear to be mediated by impairments in inhibitory control. One explanation of this null finding is that inhibitory control may not be the central route through which rDLPFC exerts influence over alcohol consumption, and that other mechanisms (e.g., craving; Rose & Grunsell, 2008 or motivation; Rose et al., 2010) might play a more determinant role. Whilst not acting as a direct mediator, our findings may therefore indicate that inhibitory control acts via a different route, possibly as a “brake” on other cognitive and psychological mechanisms. For example, inhibitory control may moderate processes, such as automatic approach tendencies (Wiers et al., 2007) and implicit associations (Houben & Wiers, 2008). Nevertheless, this interpretation is merely speculative and future research with greater power is recommended to determine the extent to which inhibitory control is the primary mechanism by which the rDLPFC exerts influence over alcohol consumption, and the degree to which other cognitive processes may play a role.

Several limitations need to be borne in mind when considering current findings. First, the within-participants design limited our ability to analyze moderation although the sample size was in line with similar work (Lowe et al., 2018). Second, to prevent procedural signaling (Davies & Best, 1996) during the bogus taste task, we did not take measures of subjective craving or motivations to drink. This precludes our ability to assess the extent to which inhibitory control may exert a moderating influence. Third, the current study delivered SST shortly after stimulation to ensure that both the SST and the bogus taste task were conducted within appropriate time frames for effects of cTBS to be observed (~35-40 minutes). However, it is worth noting the findings from Huang et al. (2005), which suggest that the peak effects of 600 pulse cTBS occur at around 14-40 minutes poststimulation. Considering these previous findings, the null findings with regards to mediation in the current study warrant future investigations with longer delays prior to the delivery of cognitive tasks if procedural/technological advances make this feasible. Fourth, the current research used a student sample. University students are immersed in a heavy drinking culture (Borsari & Carey, 2001; Karam, Kypri, & Salamoun, 2007; Knight et al., 2002), and it is possible that findings may not generalize to other populations. Finally, the small sample size of the current study may be incompatible with detecting a small mediational effect, with post hoc power analysis suggesting that a sample of 200 may be required to detect an effect. However, it is worth noting that to our knowledge to date no such study testing the relationship between fluctuations in inhibitory control and subsequent alcohol consumption meet these power analysis requirements (Field & Jones, 2017, *N* = 81; Weafer & Fillmore, 2008, *N* = 26), and the current sample size is comparative with other TMS studies (Lowe et al., 2018: *N*’s = 7-40). In view of the amount of time required to conduct this kind of research, it may prudent for researchers to collaborate via multisite studies to address power concerns (Button et al., 2013).

In conclusion, the current study represents the first attempt to apply TMS to the rDLPFC to examine the resulting effect on actual alcohol consumption. Results point to the important role of this brain structure in shaping drinking behaviour as well as driving inhibitory control. However, inhibitory control was not found to mediate the observed association between stimulation of the rDLPFC and alcohol consumption, although future investigations with more highly powered designs could fruitfully revisit this hypothesis. Overall, our findings highlight that further research appears warranted to unpick the nuanced ways in which the rDLPFC and inhibitory control shape behaviours, which require the exertion of a degree of self-control.
